# MTHFR C677T, Prothrombin G20210A, and Factor V Leiden (G1691A) Polymorphism and Beta-Thalassemia Risk: A Meta-Analysis

**DOI:** 10.7759/cureus.10743

**Published:** 2020-09-30

**Authors:** Nitu Nigam, Prithvi K Singh, Monica Agrawal, Sanjay Nigam, Harish Gupta, Shailendra Saxena

**Affiliations:** 1 Center for Advance Research (Cytogenetics Lab), King George's Medical University, Lucknow, IND; 2 Center for Advance Research (Cytogenetic Lab), King George's Medical University, Lucknow, IND; 3 Obstetrics and Gynaecology, King George's Medical University, Lucknow, IND; 4 Pathology, Saraswati Medical College, Unnao, IND; 5 Medicine, King George's Medical University, Lucknow, IND; 6 Center for Advance Research, King George's Medical University, Lucknow, IND

**Keywords:** gene polymorphism, mthfr, prothrombin, factor v leiden, β-thalassemia

## Abstract

Background

Beta (β)-thalassemia major patients frequently suffer from many vascular problems. Thrombophilia is a blood disorder that comprises imbalances in the blood coagulating factor due to ecological and hereditary components. Previous evidence shows that thrombosis is the commonest risk in beta-thalassemia patients. Several studies have examined that MTHFR C677T, prothrombin G20210A (PT G20210A), and Factor V Leiden G1691A (FVL G1691A) polymorphism play a crucial role in the development of β-thalassemia major, yet the result was questionable and uncertain. Therefore, in this study, we executed the correlation between these gene polymorphisms with β-thalassemia major patients.

Methods

Suitable keywords were used to search related articles in PubMed, Google Scholar, and Web of Science. In this random-effects meta-analysis, we analyzed the odds ratio (OR) for the estimation of risk.

Results

A total of nine research articles with 645 β-thalassemia major patients and 989 healthy controls were incorporated in this meta-analysis. The pooled OR was assessed in MTHFR C677T, PT G20210A, and FVL G1691A polymorphism. This random-effects meta-analysis demonstrated that MTHFR C677T, PT G20210A, and FVL G1691A gene polymorphism did not significantly associate with β-thalassemia major. Moreover, the heterogeneity was significantly found in genotype CC vs CT+TT C677T (I^2^=61%) and allele C vs T (I^2^=71%) of MTHFR and genotype GG vs GA (I^2^=95%), GG vs GA+AA (I^2^=95%), GA vs GG+AA (I^2^=95%), and allele G vs A (I^2^=93%) of FVL G1691A.

Conclusion

The results of this meta-analysis show that MTHFR C677T, prothrombin G20210A, and Factor V Leiden (G1691A) gene polymorphism are not a risk factor for β-thalassemia major.

## Introduction

Beta (β)-thalassemia major is a single genetic disease affected by mutations in β-globin genes. These mutation causes a complete deficiency or reduces the formation of β-globin chains [1]. This disease is an incredible social medical issue in India, where the carrier rate was 5%-7%, and globally, it was 4.4% [2-3]. β-thalassemia comprises a wide assortment of diseases in three stages, i.e. minor, intermedia, and major, in which major is a severe blood transfusion-dependent disorder [4].

Highly thromboembolic events have been associated with β-thalassemia, which leads to the thrombosis of arterial or venous blood [5-6]. Various multicenter studies demonstrated the incidence of thromboembolic events, ranging from 1.1% to 5.3%, which were observed in β-thalassemia [7-9]. Till now, the process of hypercoagulability is not clear. Previously, various studies reported that the hypercoagulability in β-thalassemia patients is associated with learned risk factors, including iron overload, hypothyroidism, splenectomy, abnormal liver function, platelet abnormalities, cardiopulmonary abnormalities, and reduced levels of natural anticoagulants [10-12]. Moreover, a study reported that methylenetetrahydrofolate reductase (MTHFR), prothrombin (PT), and Factor V Leiden (FVL) gene polymorphism were predisposing factors for thromboembolic manifestations in β-thalassemia [10].

MTHFR C667T gene polymorphism altered the function of enzymes and increased the risk of thrombosis [13]. A previous study reported that inherited thrombophilic mutations, such as MTHFR C677T, were significantly associated with hyperhomocysteinemia [13-14]. FVL G1691A polymorphism opposes stimulated protein C and increases the incidence of thrombosis. The most predominant acquired FVL gene affects thrombosis with the segregation of autosomal dominant genes. The heterozygous mutation of FVL G1691A gene polymorphism increases the incidence of thromboembolic events from 3% to 5%, whereas homozygous mutation is significantly higher disposed to thrombosis (50%-100%) [15-18]. The 3’UTR of PT G20210A (PT G20210A) polymorphism was significantly correlated with a high risk of thrombosis [15-20].

Various epidemiological studies have explored the associations of the MTHFR C677T, PT G20210A, and FVL G1691A gene polymorphism with β-thalassemia. Still, the outcomes of the studies were conflicting and indecisive. In light of the above evidence, we executed this meta-analysis of accessible articles connecting MTHFR C677T, PT G20210A, and FVL G1691A gene polymorphism to the risk of β- thalassemia.

## Materials and methods

Setting and study design

This meta-analysis study was done in the Center for Advance Research (CFAR), Cytogenetics Lab, King George’s Medical University, Lucknow.

Identification and methods: search and selection of studies

β-thalassemia related articles were independently recognized by investigators from PubMed, Google, and Web of Science. The search terms were as follows: (MTHFR C677T OR Methylenetetrahydrofolate Reductase C677T OR Prothrombin G20210A OR PT G20210A OR PRTG20210A OR Factor V Leiden G1691A OR FVL G1691A AND (polymorphism OR Single nucleotide gene polymorphism OR SNP OR allele OR genotype) AND (thalassemia OR beta-thalassemia OR β- thalassemia OR beta-thalassemia major OR β- thalassemia major OR β- TM). Moreover, the references of all articles and reviews were manually searched for extra appropriate studies.

Results were restricted to MTHFR C677T, PT G20210A, and FVL G1691A gene polymorphism and β-thalassemia major in humans. A total of 780 articles were identified in PubMed, Google, and Web of Science. Inclusion criteria for the selection and inclusion of studies were: i) Case‑control study of β-thalassemia patients and controls (healthy); (2) MTHFR C677T, PT G20210A, and FVL G1691A gene polymorphism; (3) Published articles; (4) Full article in English; (5) Studies with the expression of MTHFR C677T, PT G20210A, and FVL G1691A gene, and studies on animals or in the lab were excluded. The association of MTHFR (C677T), PT (G20210A), and FVL (G1691A) gene with β-thalassemia and control was the main concern for the selection of studies.

Data collection

Data were extracted by authors individually from articles text, figures, or tables in each published article. The above-mentioned details were collected from individual studies, i.e., types of SNP, the technique for polymorphism, and the number of patients and controls (Table 1).

**Table 1:** Characteristics/details of included studies for meta-analysis

Study/Year	Types of SNP	Country	Cases (β-thalassemia)	Controls	Methods
Al-Sweedan et al., 2009	MTHFR C677T, Prothrombin G20210A, Factor V Leiden G1691A	Jordan	100	100	PCR-RFLP
Mustafa et al., 2009	MTHFR C677T	Kuwait	50	50	PCR-RFLP
Rania et al., 2016	MTHFR C677T	Eastern Algeria	8	10	PCR-RFLP
Abd-Elmawla et al., 2016	MTHFR C677T	Egypt	66	66	PCR-RFLP
Rahimi et al., 2008	MTHFR C677T, Prothrombin G20210A, Factor V Leiden G1691A	Iran	151	180	PCR-RFLP
Bagher et al., 2017	MTHFR C677T, Prothrombin G20210A, Factor V Leiden G1691A	Iran	158	104	PCR-RFLP
Pandey et al., 2012	MTHFR C677T, Prothrombin G20210A, Factor V Leiden G1691A	India	75	297	PCR-RFLP
Nefissi et al., 2018	MTHFR C677T, Prothrombin G20210A	Tunisia	4	64	PCR-RFLP
Samarah et al., 2018	Prothrombin G20210A	Palestine	33	118	PCR-RFLP

Data synthesis

The data were obtained from every study: title of the article, name of the principal author’s, journal name, date of publication, name of nation, and strategy utilized for genotyping.

Statistical analysis

RevMan 5.3 (Review Manager version 5.3) of the Cochrane Collaboration, London, United Kingdom, was used for statistical interpretation. The odds ratio (OR) was used for risk assessment. In addition, the heterogeneity among studies was estimated by the Q test with p-values <0.05 and >50% I2 was considered significant. Initially, the associations-based studies were analyzed; after that, the subgroup examinations by SNP type were accomplished to find the SNP type-specific impacts.

## Results

Search results

**Figure 1:**
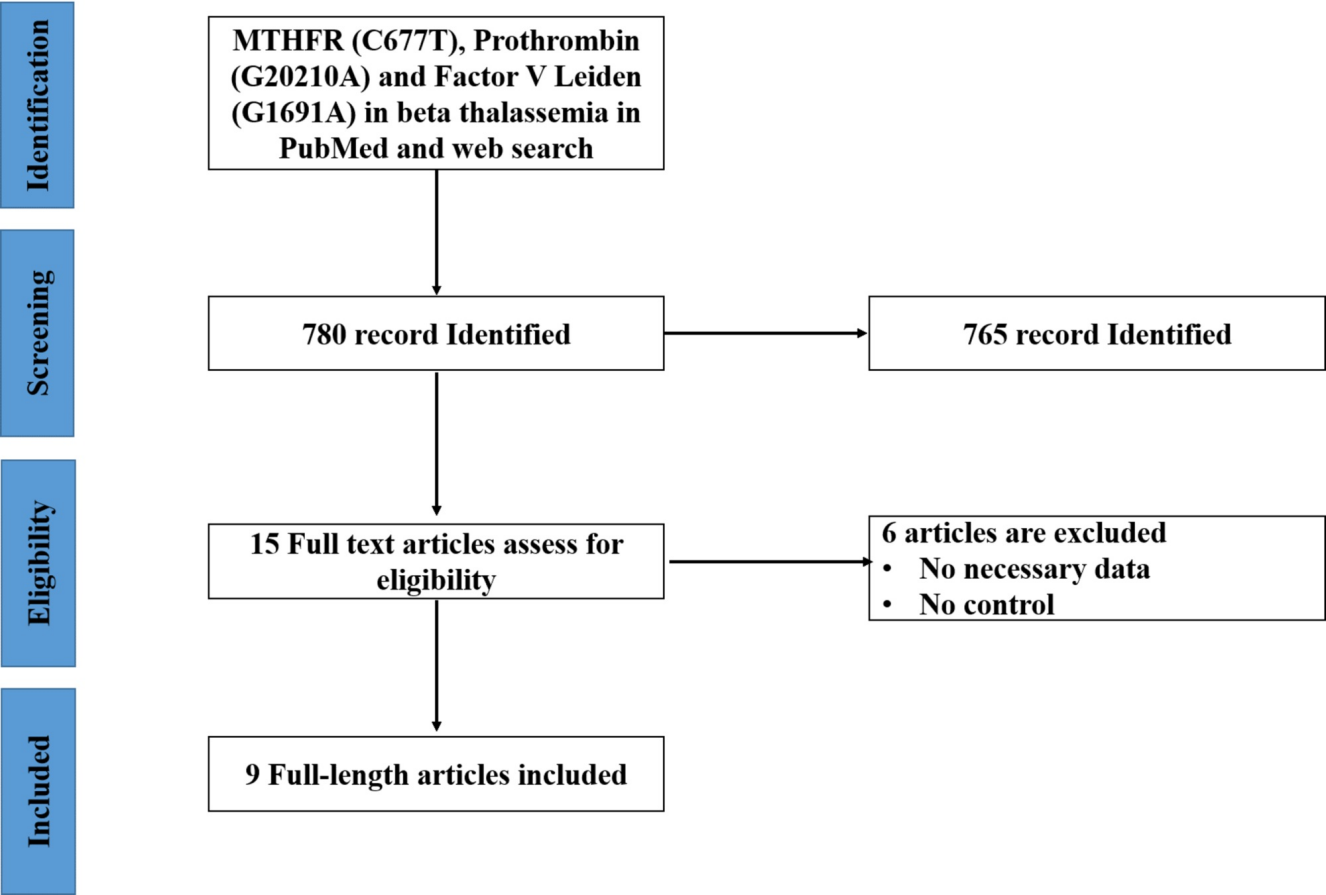
Flow diagram of article searching, screening, eligibility, and included or selection process

The search outcomes of published articles have been shown in Figure 1. Initially, 780 pieces of literature were searched and saved. After screening the title and abstracts of the articles, of a total of 765 pieces of literature records were excluded from this meta-analysis due to the non-relevance with the theme, duplicated works, and review articles. Furthermore, six published articles were also left out after the screening of record abstracts. After scanning of abstracts and titles, 15 articles were identified for full-text scrutiny. Numerous studies were excluded due to the unavailability of necessary data and controls. Hence, a total of nine studies encompassing 645 β-thalassemia major patients and 989 healthy controls were included in the meta-analysis. Finally, nine articles [14,21-28], eight studies focusing on polymo­phism of MTHFR C677T, six studies on prothrombin G20210A, and four studies on Factor V Leiden G1691A were considered for meta‑analysis (Figure 1).

MTHFR C677T gene polymorphism and the risk of β-thalassemia

**Figure 2:**
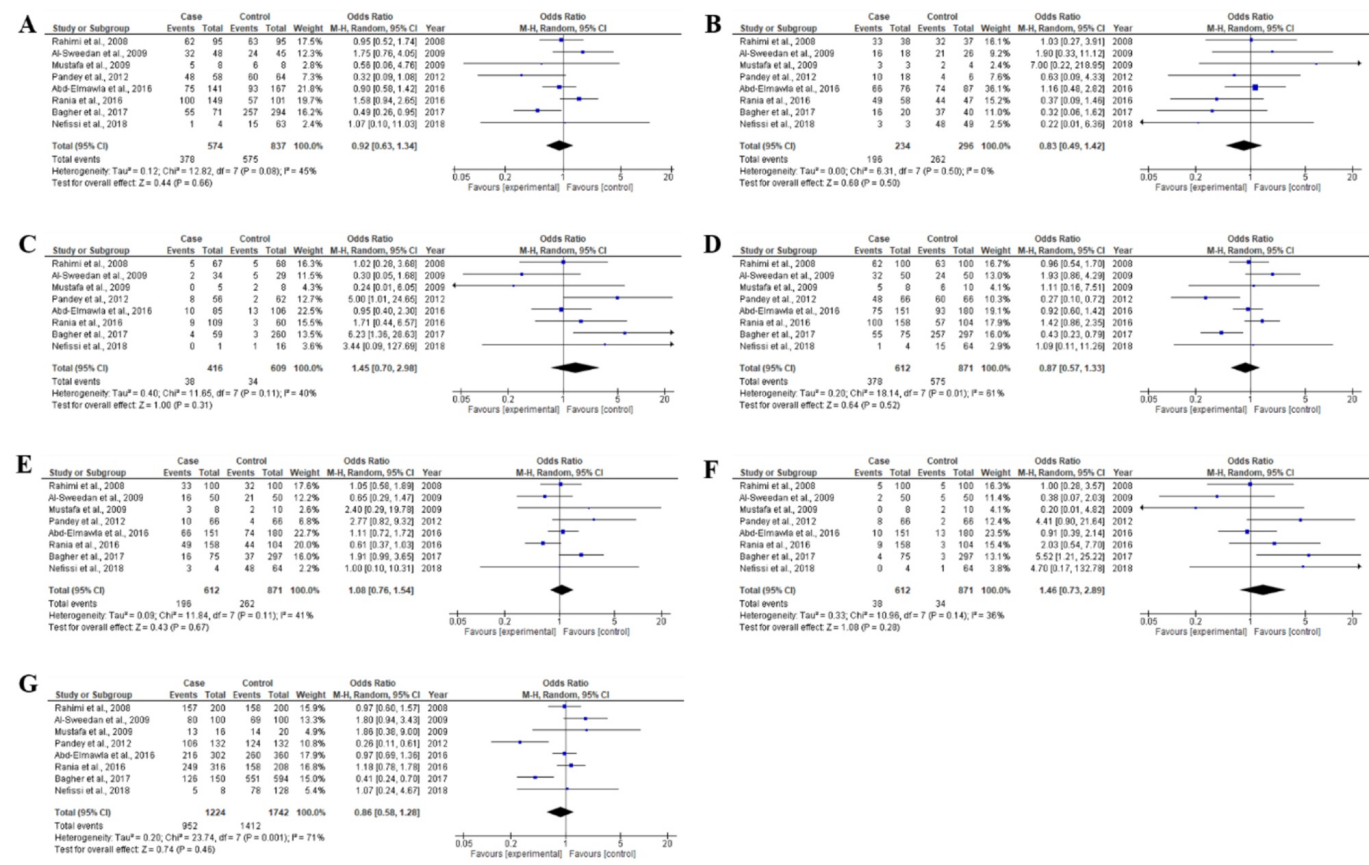
Forest plots of association between MTHFR C677T polymorphism and β-thalassemia for all genotype and allele frequencies (A) CC vs. CT genotype, (B) CT vs. TT genotype, (C) TT vs. CC genotype, (D) CC vs. CT+TT genotype, (E) CT vs. CC+TT genotype, (F) TT vs. CC+CT genotype, (G) C vs. T allele The squares and horizontal lines parallel to the OR and 95% CI for study. The squares area shows the weight (inverse of the variance). The diamond area shows the overall OR and 95% CI. MTHFR, methylenetetrahydrofolate reductase; OR, odds ratio; CI, confidence interval

For MTHFR C677T polymorphism, a total of eight studies, including 612 β-thalassemia patients and 871 healthy controls were examined (Table 1). Deviations from Hardy-Weinberg Equilibrium (HWE) were observed in only one study [28] while the other eight studies were in agreement with HWE (Table 2). Random effects meta-analysis demonstrated that MTHFR C677T gene polymorphism did not found significantly associated with β-thalassemia risk [CC vs CT: OR 0.92, CI(0.63-1.34), p=0.68; CT vs TT: OR 0.83, CI(0.49-1.42), p=0.50; TT vs CC: OR1.45 CI(0.70-2.98), p=0.31; CC vs CT+TT: OR 0.87, CI(0.57-1.33), 0.52; CT vs TT+CC: OR 1.08, CI(0.76-1.54), p=0.67; TT vs CC+CT: OR 1.46, CI(0.73-2.89), p=0.28 and C vs T: OR 0.86, CI(0.58-1.28)], as shown in Table 3 and Figure 2. Moreover, the heterogeneity was significantly found in genotype CC vs CT+TT (I2=61%) and allele C vs T (I2=71%) analyzed in this meta-analysis (Table 3).

**Table 2:** Distributions of genotypes and allele frequencies of MTHFR (C677T), prothrombin (G20210A), and Factor V Leiden (G1691A) in β-thalassemia cases and controls

Study	Cases					Controls					
	Genotype			Allele		Genotype			Allele		
MTHFR C677T											
	CC	CT	TT	C	T	CC	CT	TT	C	T	
Al-Sweedan et al., 2009	62	33	5	157	43	63	32	5	158	42	
Mustafa et al., 2009	32	16	2	80	20	24	21	5	69	31	
Rania et al., 2016	5	3	0	13	3	6	2	2	14	6	
Abd-Elmawla et al., 2016	48	10	8	106	26	60	4	2	124	8	
Rahimi et al., 2008	75	66	10	216	86	93	74	13	260	100	
Bagher et al., 2017	100	49	9	249	67	57	44	3	158	50	
Pandey et al., 2012	55	16	4	126	24	257	37	3	551	43	
Nefissi et al., 2018	1	3	0	5	3	15	48	1	78	50	
Prothrombin G20210A											
	GG	GA	AA	G	A	GG	GA	AA	G	A	
Rahimi et al., 2008	149	2	0	300	2	174	6	0	354	6	
Al-Sweedan et al., 2009	94	6	0	198	6	95	5	0	195	5	
Nefissi et al., 2018	16	0	0	32	0	57	7	0	121	7	
Bagher et al., 2017	158	0	0	316	0	103	1	0	207	1	
Samarah et al., 2018	29	4	0	62	4	112	6	0	230	6	
Pandey et al., 2012	75	0	0	150	0	297	0	0	594	0	
Factor V Leiden G1691A											
	GG	GA	AA	G	A	GG	GA	AA	G	A	
Rahimi et al., 2008	143	7	1	293	9	175	5	0	355	5	
Al-Sweedan et al., 2009	78	21	1	177	23	19	80	1	118	82	
Bagher et al., 2017	151	7	0	309	7	98	3	2	199	7	
Pandey et al., 2012	66	8	1	140	10	289	8	0	586	8	

**Table 3:** Subgroup analyses for MTHFR C677T polymorphism and β-thalassemia *=Significant

Variable	Main effects			Heterogeneity			
	OR (95% CI)	z Score	P-value	Tau^2^	Chi^2^	P-value	I^2^ statistic
CC vs CT	0.92 (0.63-1.34)	0.44	0.68	0.12	12.82	0.08	45%
CT vs TT	0.83 (0.49-1.42)	0.68	0.50	0.0	6.31	0.50	0%
TT vs CC	1.45 (0.70-2.98)	1.00	0.31	0.40	11.65	0.11	40%
CC vs CT+TT	0.87 (0.57-1.33)	0.64	0.52	0.20	18.14	0.01^*^	61%
CT vs TT+CC	1.08 (0.76-1.54)	0.43	0.67	0.09	11.84	0.11	41%
TT vs CC+CT	1.46 (0.73-2.89)	1.08	0.28	0.33	10.96	0.14	36%
C vs T	0.86 (0.58-1.28)	0.74	0.46	0.20	23.74	0.001^*^	71%

Prothrombin G20210A gene polymorphism and the risk of β-thalassemia

**Figure 3:**
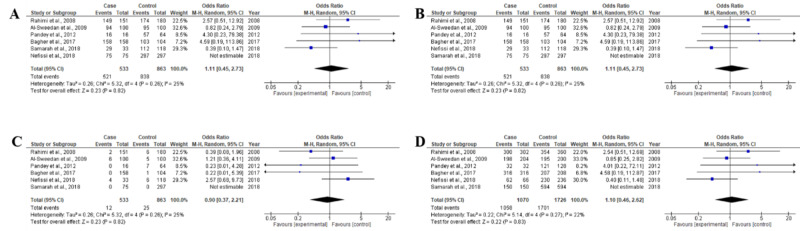
Forest plots of association between prothrombin G20210A polymorphism and β-thalassemia for all genotype and allele frequencies (A) GG vs. GA genotype, (B) GG vs. GA+AA genotype, (C) GA vs. GG+AA genotype, (D) G vs. A allele The squares and horizontal lines parallel the OR and 95% CI for the study. The square areas show the weight (inverse of the variance). The diamond area shows the overall OR and 95% CI. OR, odds ratio; CI, confidence interval

For prothrombin G20210A polymorphism, a total of six studies, including 521 β-thalassemia patients and 863 healthy controls were examined (Table 1). Deviations from HWE were not observed in any studies, all six studies were in agreement with HWE (Table 2). This random-effects meta-analysis demonstrated that prothrombin G20210A gene polymorphism was not found significantly associated with β-thalassemia risk [GG vs GA: OR 1.11, CI(0.45-2.73), p=0.82; GG vs GA+AA: OR 1.11, CI(0.45-2.73), p=0.82; GA vs GG+AA: OR 0.90, CI(0.37-22.21), p=0.82; and G vs A: OR 1.10, CI(0.46-2.62), p=0.83], as shown in Table 4 and Figure 3. Moreover, heterogeneity was also not significantly found in genotype and allele prothrombin G20210A analyzed in this meta-analysis (Table 4).

**Table 4:** Subgroup analyses for prothrombin G20210A polymorphism and β-thalassemia

Variable	Main effects			Heterogeneity			
	OR (95% CI)	z Score	P-value	Tau^2^	Chi^2^	P-value	I^2^ statistic
GG vs GA	1.11 (0.45-2.73)	0.23	0.82	0.26	5.32	0.26	25%
GA vs AA	-	-	-	-	-	-	-
AA vs GG	-	-	-	-	-	-	-
GG vs GA+AA	1.11 (0.45-2.73)	0.23	0.82	0.26	5.32	0.26	25%
GA vs GG+AA	0.90 (0.37-22.21)	0.23	0.82	0.26	5.32	0.26	25%
AA vs GG+GA	-	-	-	-	-	-	-
G vs A	1.10 (0.46-2.62)	0.22	0.83	0.22	5.14	0.27	22%

Prothrombin G20210A gene polymorphism and the risk of β-thalassemia

For prothrombin G20210A polymorphism, a total of six studies, including 521 β-thalassemia patients and 863 healthy controls were examined (Table 1). Deviations from HWE were not observed in any studies, all six studies were in agreement with HWE (Table 2). This random-effects meta-analysis demonstrated that prothrombin G20210A gene polymorphism was not found to be significantly associated with β-thalassemia risk [GG vs GA: OR 1.11, CI(0.45-2.73), p=0.82; GG vs GA+AA: OR 1.11, CI(0.45-2.73), p=0.82; GA vs GG+AA: OR 0.90, CI(0.37-22.21), p=0.82; and G vs A: OR 1.10, CI(0.46-2.62), p=0.83] as shown in Table 4 and Figure 3. Moreover, heterogeneity was also not significantly found in the genotype and allele prothrombin G20210A analyzed in this meta-analysis (Table 4).

Factor V Leiden G1691A gene polymorphism and the risk of β-thalassemia

**Figure 4:**
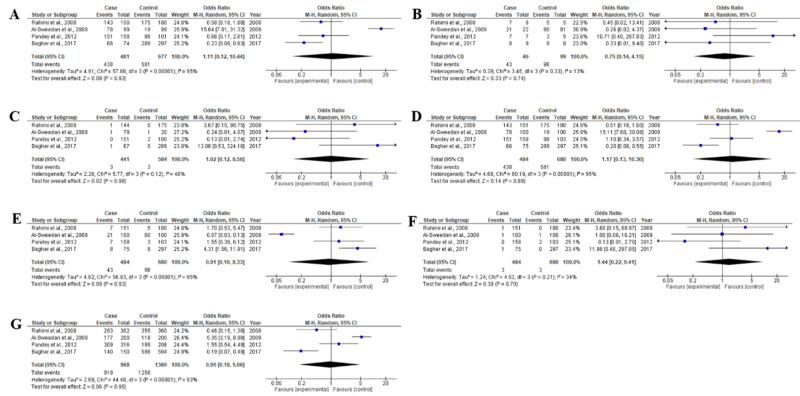
Forest plots of association between Factor V Leiden G1691A polymorphism and β-thalassemia for all genotype and allele frequencies (A) GG vs. GA genotype, (B) GA vs. AA genotype, (C) AA vs. GG genotype, (D) GG vs. GA+AA genotype, (E) GA vs. GG+AA genotype, (F) AA vs. GG+GA genotype, (G) G vs. A allele The squares and horizontal lines are parallel to the OR and 95% CI for the study. The squares area shows the weight (inverse of the variance). The diamond area shows the overall OR and 95% CI. OR, odds ratio; CI, confidence interval

For Factor V Leiden G1691A polymorphism, a total of four studies, including 484 β-thalassemia patients and 681 healthy controls, were examined (Table 1). Deviations from HWE were observed in two studies [25,27] while the other two studies were in agreement with HWE (Table 2). This random-effects meta-analysis demonstrated that prothrombin G20210A gene polymorphism was not found to be significantly associated with β-thalassemia risk [GG vs GA: OR 1.11, CI(0.12-10.44), p=0.93; GA vs AA: OR 0.75, CI(0.14-4.15), p=0.74; AA vs GG: OR 1.02, CI (0.2-8.58) p=0.98; GG vs GA+AA: OR 1.17, CI (0.13-10.30), p=0.89; GA vs GG+AA: OR 0.91, CI (0.10-8.33), p=0.93; AA vs GG+GA: OR 1.44, CI (0.22-9.41), 0.70 and G vs A: OR 0.95, CI (0.18-5.06), p=0.95] as shown in Table 5 and Figure 4. Moreover, heterogeneity was significantly found in genotype GG vs GA (I2=95%), GG vs GA+AA (I2=95%), GA vs GG+AA (I2=95%), and allele G vs A (I2=93%) analyzed in this meta-analysis (Table 5).

**Table 5:** Subgroup analyses for Factor V Leiden G1691A polymorphism and β-thalassemia *=Significant

Variable	Main effects			Heterogeneity			
	OR (95% CI)	z Score	P-value	Tau^2^	Chi^2^	P-value	I^2^ statistic
GG vs GA	1.11 (0.12-10.44)	0.009	0.93	4.91	57.66	<0.00^*^	95%
GA vs AA	0.75 (0.14-4.15)	0.33	0.74	0.39	3.45	0.33	13%
AA vs GG	1.02 (0.2-8.58)	0.02	0.98	2.26	5.77	0.12	48%
GG vs GA+AA	1.17 (0.13-10.30)	0.14	0.89	4.66	60.9	<0.00^*^	95%
GA vs GG+AA	0.91 (0.10-8.33)	0.09	0.93	4.82	56.93	<0.001^*^	95%
AA vs GG+GA	1.44 (0.22-9.41)	0.38	0.70	1.24	4.53	0.21	34%
G vs A	0.95 (0.18-5.06)	0.06	0.95	2.69	44.48	<0.001^*^	93%

## Discussion

Meta-analysis is a potent analyzing tool for accumulative data with low and small power studies. In this study, we explore the correlation of MTHFR C677T, PT G20210A, and FVL G1691A gene polymorphism with β-thalassemia major patients. Here, we meta-analyzed the published information about the polymorphism of MTHFR C677T of 612 β-Thalassemia major patients from eight published studies and, particularly, the changes in allele and genotype occurrences of MTHFR C677T gene polymorphism in patients as compared with controls. We also meta-analyzed the published data about the polymor­phism of PT G20210A and FVL G1691A of 521 and 484 β-thalassemia major patients from six and four studies, respectively, and compared the genotype and allele frequencies of PT G20210A and FVL G1691A polymorphism in patients as compared with controls.

In this random-effects meta-analysis, we did not find an association between MTHFR C677T, PT G20210A, and FVL G1691A gene polymorphism with β-Thalassemia major patients as compared to controls. Overall, it can be concluded from general and subgroup analyses that the MTHFR C677T, PT G20210A, and FVL G1691A gene polymorphisms may not play an important role in the pathogenesis of a thromboembolic event. Still, there is not a sufficient amount of relevant studies to give a safe and good assumption. In the future, however, more well-designed studies with a bigger sample size and metacentric studies will be necessary to validate the current outcomes.

In meta-analyses, heterogeneity is one of the significant factors. In this study, heterogeneity was significantly observed in genotype CC vs CT+TT (I2=61%) and allele C vs T (I2=71%) of MTHFR C677T polymorphism and genotype GG vs GA (I2=95%), GG vs GA+AA (I2=95%), GA vs GG+AA (I2=95%), and allele G vs A (I2=93%) FVL G1691A gene polymorphism. However, heterogeneity was not significantly found in other genotypes of MTHFR C677T and FVL G1691A and the genotype and alleles of PT G20210A gene polymorphism.

A previous study reported that the T allele (mutant allele) frequencies of MTHFR C677T polymorphism were 21.5% and 21% in β-Thalassemia major and healthy controls, respectively. The frequency of MTHFR C677T mutations was marginally greater but not significantly different between β-thalassemia major patients and controls [25,27]. The frequencies of the allele and genotype of MTHFR C677T gene polymorphism were not significantly associated with β-thalassemia [23,25-26].

The mutation of the MTHFR C677T gene was not statistically correlated with an increased risk of thrombophilia in patients with β-thalassemia major [14,22]. Contrarily, Elmawla et al. (2016) reported that the polymorphism of the MTHFR C677T gene was statistically significantly correlated with β-thalassemia [21]. The frequencies of the mutant A allele of FVL G1691A gene polymorphism was 11.5% in β-thalassemia major and 10.5% in controls. The frequencies of G and A alleles were not significantly different between groups [25]. The incidence of the FVL G1691A gene polymorphism was found more in β-thalassemia patients but not significantly different from healthy controls. The A allele (mutant allele) frequencies of the PT G20210A mutation was 3% and 2.5% in β-thalassemia major and healthy controls, respectively; differences in between were not statistically significant [25]. Pandey et al. (2012) show that the occurrence of heterozygous PT G20210A polymorphism was not associated in patients in the β-thalassemia major and controls groups [27]. Limited studies demonstrated that the increased frequency of thrombophilic mutation has not been associated with thalassemia patients [12,29]. However, the heterozygote genotype of FVL G1691A and PT G20210A and the frequencies of recurrent thromboembolism was double in β-thalassemia patients [30]. The heterozygous and homozygous genotypes of FVL was statistically significantly correlated with the high risk of thrombosis [17].

When interpreting the meta-analysis outcomes, various limitations should be considered. First of all, the appropriate studies were limited in number. To limit the conceivable outcomes of distribution bias, published articles were incorporated. Thereafter, the present results were based on unadjusted appraisals. Finally, our analyses did not reflect the possibility of linkage disequilibrium and interaction between gene-gene or SNP-SNP.

## Conclusions

The results of the present meta-analysis suggest that the genotype and allele frequencies of MTHFR C677T, prothrombin G20210A (PT G20210A), and Factor V Leiden G1691A (FVL G1691A) gene polymorphism were not significantly correlated with the risk for the development of β-thalassemia major. However, the outcomes of this meta-analysis were based on mutation (single gene polymorphism), and significant heterogeneity was likewise identified. Therefore, the outcomes of the current study should be interpreted and need further well-designed studies.
